# Aqueous Dispersions of Silica Stabilized with Oleic Acid Obtained by Green Chemistry

**DOI:** 10.3390/nano6010009

**Published:** 2016-01-05

**Authors:** Cristina Lavinia Nistor, Raluca Ianchis, Marius Ghiurea, Cristian-Andi Nicolae, Catalin-Ilie Spataru, Daniela Cristina Culita, Jeanina Pandele Cusu, Victor Fruth, Florin Oancea, Dan Donescu

**Affiliations:** 1 R & D National Institute for Chemistry and Petrochemistry, Spl. Independentei 202, 6th District, 060021 Bucharest, Romania; lc_nistor@yahoo.com (C.L.N.); ghiurea@yahoo.com (M.G.); ca_nicolae@yahoo.com (C.-A.N.); catalin_spataru2004@yahoo.com (C.-I.S.); ddonescu@chimfiz.icf.ro (D.D.); 2 “Ilie Murgulescu” Institute of Physical Chemistry of Romania Academy, Spl. Independentei 202, 6th District, 060021 Bucharest, Romania; dculita@icf.ro (D.C.C.); jeaninamirea@yahoo.com (J.P.C.); vfruth@icf.ro and florino@ping.ro (V.F.)

**Keywords:** nanosilica, hydrophobic, octadecyltrimethoxysilane, oleic acid, sodium silicate

## Abstract

The present study describes for the first time the synthesis of silica nanoparticles starting from sodium silicate and oleic acid (OLA). The interactions between OLA and sodium silicate require an optimal OLA/OLANa molar ratio able to generate vesicles that can stabilize silica particles obtained by the sol-gel process of sodium silicate. The optimal molar ratio of OLA/OLANa can be ensured by a proper selection of OLA and respectively of sodium silicate concentration. The titration of sodium silicate with OLA revealed a stabilization phenomenon of silica/OLA vesicles and the dependence between their average size and reagent’s molar ratio. Dynamic light scattering (DLS) and scanning electron microscopy (SEM) measurements emphasized the successful synthesis of silica nanoparticles starting from renewable materials, in mild condition of green chemistry. By grafting octadecyltrimethoxysilane on the initial silica particles, an increased interaction between silica particles and the OLA/OLANa complex was achieved. This interaction between the oleyl and octadecyl chains resulted in the formation of stable gel-like aqueous systems. Subsequently, olive oil and an oleophylic red dye were solubilized in these stable aqueous systems. This great dispersing capacity of oleosoluble compounds opens new perspectives for future green chemistry applications. After the removal of water and of the organic chains by thermal treatment, mesoporous silica was obtained.

## 1. Introduction

Mesoporous materials have generated a great deal of interest over the last two decades. This interest was motivated by their use in catalysis, followed by finding new areas of application, such as: chromatography, membranes, sensors, biotechnology, nanomedicine, *etc.* [1,2,3,4,5,6,7,8]. A very important goal in the synthesis of these materials was to fundamentally explain the factors that affect their final properties. Mostly, these types of structures are obtained by sol-gel processes, generated in the presence of amphiphilic substances that act as *supramolecular structure directing agents*—SDAs. For silica materials, SDAs are usually cationic or anionic surfactants. They provide a cooperative assembly of inorganic species through an electrical self-assembly pathway or an electrically neutral pathway [1,2,8].

The global energy crisis, intense concern over polluting processes, and increased interest in separation, drug delivery and sensing have jumpstarted the search for environmentally friendly materials based on natural and renewable raw materials [8]. Since the sol-gel processes occur mostly at room temperature, in a clean aqueous medium in the presence of ethanol from plant resources, directions for green chemistry synthesis can be established. These directions can also provide cost savings for future productions since SDAs derived from renewable resources and a silica precursor from a large natural mineral deposit, such as quartz from sand, will be inexpensive [8]. Thus, at temperatures above 1000 °C, sand reacts with sodium hydroxide or sodium carbonate and generates water-soluble sodium silicate [8]. These aqueous solutions are cheap raw materials that in the presence of cationic or nonionic surfactants generate mesoporous silica [8,9,10,11,12,13,14]. A very important phenomenon concerning sodium silicate aqueous solutions refers to the association degree of the cationic SDA that does not change until the occurrence of silica precipitate [14]. The increase of the quantity of silica precipitate will determine a progressive increase of the bounded SDA. Interactions between silica precipitate and the cationic SDA occur similarly to the ones between polyelectrolytes and surfactants [14].

The surfactants obtained from renewable sources are efficient in the synthesis of mesoporous silica [8]. Among the diversity of SDAs that can be prepared from renewable sources, the ones coming from vegetable oils are very promising. This conclusion is based on the abundance of vegetable oils that can be extracted from oleaginous plant seeds [8]. The price for fatty acids obtained from saponification of vegetable triglycerides is smaller than other SDAs [8]. Bio-derived oleyl surfactants, oleyl amine, *N*-oleyl 1,3-diamino propane were proved to be efficient SDAs in the obtaining of mesoporous silicas through sol-gel processes of tetraethoxysilane [15]. Due to their double bond, the conformation of oleyl hydrocarbon chains can be modified depending on the temperature and polarity of the medium, a phenomenon that affects the silica particle’s pore size [15].

Thus, we evaluated the possibility of obtaining mesoporous silica directly from sodium silicate and oleic acid (OLA) without any chemical modification. By doing this, biodegradable hybrids starting from unmodified vegetable resources could be obtained. After the OLA adsorption, through a very efficient stirring (Pickering emulsions) [16], the hydrocarbon emulsions can be stabilized into a self-assembled liquid-crystalline structure from the resulting hydrophobic silica particles.

The main objective of this study is to obtain mesoporous silica from natural raw materials (sodium silicate from sand and OLA from saponified vegetable oils). When the two components react, the sodium ion will neutralize a fraction of OLA, causing the formation of its alkaline salt/soap (OLANa). After this neutralization, the pH will decrease, allowing the silica to be generated. Depending on the ratio between the two reactants (sodium silicate and OLA), different proportions of oleic acid (OLA) and its sodium salt (OLANa) coexist in the reaction medium.

In an aqueous medium, the OLA/OLANa (acid/soap) system can produce stable structures—liposomes, depending on concentration, pH and temperature [17,18,19,20,21,22,23,24]. Due to the presence of associated hydrophobic chain domains, these vesicular aggregates can be used for the encapsulation of some bioactive products, with various applications: drug delivery, cosmetics, enzyme encapsulation, food technology, *etc.* [17,18,19,25,26,27]. Moreover, the presence of mesoporous silica in the OLA/OLANasystem will allow the obtaining through a “green chemistry” process of more robust and biodegradable systems [28] from ecological raw materials.

The conditions to obtain stable aqueous dispersions that contain silica nanoparticles stabilized with oleic acid/sodium oleate (OLA/OLANa) complex are analyzed in this study. To the best of our knowledge, this system has not been previously reported. In order to elucidate how silica particlescan be producedstarting from sodiumsilicatesolutions by neutralization with OLA, we followed the investigation of the initial systems at various molar ratios of the reactants. The influence of these ratios over more complex systems, in which silica is hydrophobically modified by grafting octadecyl chains, was evaluated next. This modification was carried out by using various silica/octadecyltrimethoxysilane (ODTMOS) ratios. It is already well known that, through this process, at higher substitution degrees, the hydrophobic silica particles form themselves into lamellar associates [29]. The hydrophobic interactions between the octadecyl chains grafted on the silica and the oleyl chains from OLA/OLANa mixtures increases the stability of the systems and allows the solubilization of different oleophile additions. The stability in time of the resulted systems and their homogeneity were observed using olive oil as a model-oleofil, red-colored with a commercially available dye (Solvent Red).

## 2. Results and Discussion

### 2.1. Formation of Silica Particles from Sodium Silicate and Oleic Acid (OLA)

**Figure 1:**
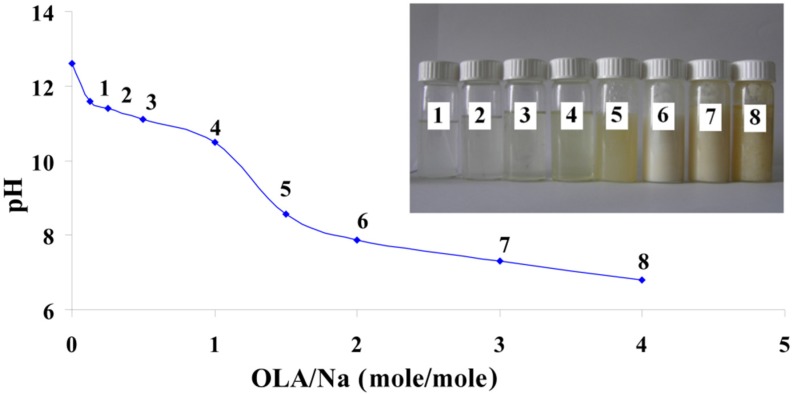
pH modification of the dispersions prepared at a fixed amount of sodium silicate and different quantities of oleic acid (OLA); **Inset Picture**: The dispersions prepared at different quantities of OLA and a fixed amount of sodium silicate (**1.** 0.125/1; **2.** 0.25/1; **3.** 0.5/1; **4.** 1/1; **5.** 1.5/1; **6.** 2/1; **7.** 3/1 and **8.** 4/1 OLA/Na).

To overcome the Kraft temperature that was previously mentioned for OLANa (the Kraft temperature for 1 wt % of OLANa in water is about 23 °C and for 35 wt % is 33 °C), the titration of sodium silicate with OLA (2.2.1) was performed at 40 °C [30]. Figure 1 presents the pH modification of these mixtures depending on the OLA/Na ratio.

The decrease of the pH value with the increase of the OLA concentration is similar to previously published results, where the alkaline salts of OLA are titrated with acid [17,18,19,20,21,22,23,31,32,33]. The systems are transparent up to a ratio of OLA/Na = 1/1 and exhibits a slow decrease of the pH value (inset Figure 1). In these systems, OLA is found in the OLANa form and forms micelles. As the molar ratio of OLA/Na increased from 0.125/1 to 4/1, the systems became opaque and stable in time. In this pH range, OLA and OLANa form compounds with complex structures (bilayered vesicles). In good agreement with previously published results, the occurrence of system opacity at OLA/Na = 1.5/1 proves that the driving force of phase segregation is not only represented by the occurrence of COO-HOOC dimer, but also by the hydrophobic interaction between the oleyl chains [32,33].

**Figure 2:**
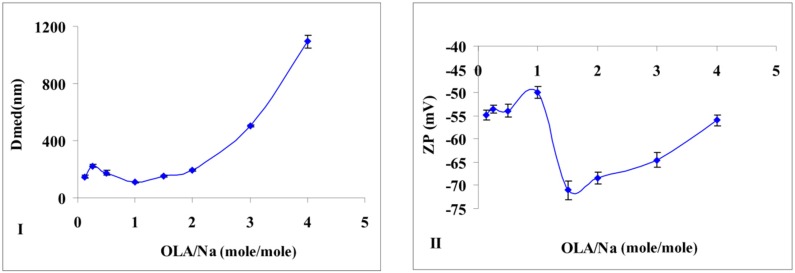
(**I**) Size modification of the dispersions prepared at a fixed amount of sodium silicate and different quantities of OLA; (**II**) Zeta potential modification of the dispersions prepared at a fixed amount of sodium silicate and different quantities of OLA (Dmed represents the medium/average diameter recorded for the prepared silica dispersions).

Additional information is provided by the particle size measurements of the obtained silica (Figure 2I). If no major modification of particle size is noticed in the interval of OLA/Na = 0.125/1 ÷ 1.5/1, after a ratio of 2/1, the dimensions for the newly formed aggregates increases tremendously (Figure 2I). In the transparent mixtures, in which OLANa exists in a micellar state, the interactions between the surfactant and silica are not very strong. The analysis of the OLANa bonded to the preformed silica particles proved only a partial adsorption at the interface [16].

The effect of this adsorption was observed even in the particle size distribution. Until a ratio of OLA/Na = 2/1, the size distribution diagrams presented a bimodal distribution (Figure S1). The particle diameters (Dmed) represented in Figure 2I are average values, but the samples obtained until a value of OLA/Na = 1/1 showed also a population of small particles (25–30 nm). Considering the synthesis conditions (pH < 11.6), sodium silicate was able to generate silica particles. Above a ratio of OLA/Na = 2/1 the particle size distribution was monomodal due to the increased aggregation capacity of OLA/OLANa mixtures.

**Figure 3:**
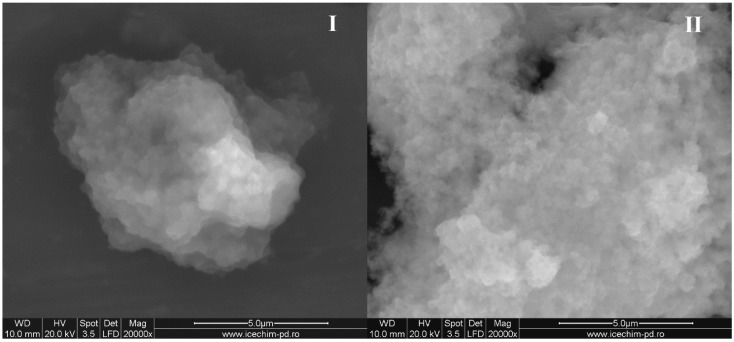
Scanning electron microscopy (SEM) pictures of the samples obtained at (**I**) 1.5/1 (sample No.5) and (**II**) 3/1 OLA/Na (sample No.7).

SEM images were in good agreement with the obtained DLS data (Figure 3). Silica nanoparticles stabilized by OLA are shown as aggregates, their medium size subsequently modified with OLA concentration.

If the OLA/Na ratio was increased, the absolute value of zeta potential decreased (Figure 2II). A break of continuity at a ratio OLA/OLANa = 1.5/1, when the whole mixture became opaque, was observed. The aggregation of OLA with OLANa increases due the hydrophobic associations in an acid/soap complex [32,33], and the negatively charged silica particles concentrate at the surface.

The dispersions obtained in the conditions mentioned above were stable for one year. The opacity was preserved at a ratio of OLA/OLANa = 1.5/1, even if the samples were diluted. To the best of our knowledge, the experiments analyzed in this paper prove the capacity of the OLA/OLANa complex to stabilize silica particles generated from the neutralization of sodium silicate with fatty acid for the first time.

### 2.2. Dispersions of Octadecyltrimethoxysilane (ODTMOS)Hydrophobic Silica

The synthesis method for these new materials as aqueous dispersions is mentioned in Section 3.2.2. The goal was to study the hydrophobic effect of the silica formed through the condensation of sodium silicate with ODTMOS.

It is well known that the presence of monoeoleines increases the stability of some complex structures that are formed by the interaction with OLA or OLANa [30,34]. Because these complex systems are modifying their morphology depending on the pH, respectively by the presence of the protonated or nonprotonated carboxyl group, the importance of the OLA/OLANa ratio through the modification of the initial concentration of OLA was evaluated (Table 1).

**Table 1:** Synthesis conditions and the properties of dispersions obtained with octadecyltrimethoxysilane (ODTMOS).

No.	ODTMOS/SiO_2_ (mole/mole)	OLA/Na (mole/mole)	*f*_OLA_ (mole/mole)OLA/(OLA + ODTMOS)	Properties of the Dispersion				Solid Hybrids		Final App.**
				pH	D (nm)	ZP (mV)	ΔH* (J/mole) (OLA + ODTMOS)	*T*_max_ (°C)	Inorg. Residue (%)	
A	1/1	2/1	0.73	7.2	188	−60.7	378	469	14	H
B	1/1	4/1	0.84	6.3	644	−60.7	580			H
C	1/1	1/1	0.54	9.2	256	−71	74			PS
D	1/1	0.5/1	0.39	10	256	−59.7	76			PS
E	1/10	2/1	0.96	7.3	516	−72	261	436	16	H
F	1/1	2/1	0.73	7.03	225	−79.7	236	474	21	H OLA in EtOH
G	1/5	2/1	0.93	7.3	740	−70.2	302	439	15	H
H	0/1	2/1	1.0	7.4	171	−61	483	423	19	H
I	1/1	1/1 HCl/Na = 0.25/1	0.54	7.1	285	−68.7	54	466	27	H
J	1/1	0.5/1 HCl/Na = 0.25/1	0.39	10.2	213	−35.5	55	395	37	PS

* melting enthalpy at 10 °C for 1 mole of hydrophobic substance (OLA moles + ODTMOS moles); ** Final appearance: H-homogeneous; PS-phase separation.

The preliminary attempts were carried out with a sodium silicate solution containing 8% Na_2_O (Merck). Homogeneous aqueous dispersions were obtained at a ratio of OLA/Na > 2/1. These results indicated an increased stability induced by the acid/soap complex (OLA/OLANa) [17,18,19,20,31,32]. Since it was previously reported that sodium silicate solutions with a higher concentration of sodium induced the formation of mesoporous silica [35], our study continued with a technical product with a concentration of Na_2_O of 14.12% (Table 1). Thus, the preliminary attempts were verified by using a sodium silicate with a higher concentration of sodium. *Sample A* (OLA/Na = 2/1, Table 1), is stable and homogeneous, and the colored olive oil is well dispersed. A stable dispersion was obtained also at a ratio of OLA/Na = 4/1 (*sample B*).

When the quantity of OLA was reduced to 1/1 or 0.5/1 OLA/Na molar ratio, unstable dispersions were obtained (*samples C* and *sample D*, Table 1). In these conditions, the final pH increased (pH = 9.2 for *sample C* and pH = 10 for *sample D*) proving that the sodium salt (OLANa) was not able to stabilize the dispersion, which is in agreement with previously published data [16].

In order to verify the stabilizing effect of the OLA/OLANa complex, two additional syntheses were tested: *samples I* and *J* (Table 1). Considering that for *sample I*, half of Na ions were neutralized with HCl, the remaining ions ensured the neutralization for 50% of OLA. As a consequence, by using the OLA/OLANa = 1/1 complex, the stability of the final dispersion was ensured. If the HCl is consumed for the neutralization of Na ions, its quantity is reduced (*sample J*) and the dispersion becomes unstable. In addition, the sample’s pH turns alkaline (pH = 10.2), as in the case of *sample D*, which was also unstable.

**Figure 4:**
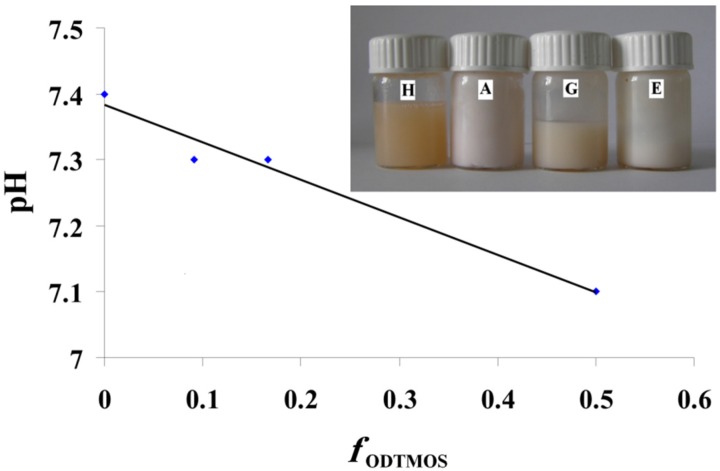
The modification of pH depending on the ODTMOS fraction; **Inset picture**: The dispersions prepared at OLA/Na 2/1 (mole/mole) and 0/1(*sample H*); 1/1 (*sample A*); 1/5 (*sample G*) and 1/10 (*sample E*) (ODTMOS/SiO_2_ (mole/mole)).

Furthermore, it was verified if the system OLA/Na = 2/1 can stabilize the silica particles functionalized with small quantities of ODTMOS (*samples E and G*) or silica particles prepared without trialkoxysilane derivate (*sample H*). All the three systems that we studied generated stable and homogeneous dispersions and all of the red-colored olive oil was entrapped in the SDA hydrophobic areas (Figure 4). It was observed that the dispersions of these samples remained stable even after 12 months after the synthesis.

An important observation was that the dispersion obtained without ODTMOS (*sample H*) was more fluid. This proves that the octadecyl groups from ODTMOS had a stabilizing effect for the OLA/OLANa complex, as in the case of monooleines [30,34]. A possible explanation is that the octadecyl chains from the silica surface can form lamellar areas [29] included in the oleyl hydrophobic areas from the OLA/OLANa mixture [32,33]. To see if these lamellar areas were affected by the method of adding the ODTMOS into the reaction mixture [29], this trialkoxysilane derivate was either dissolved in ethanol together with OLA (*sample F*) or in the aqueous phase (*sample A*). Both samples were homogeneous and showed an increased viscosity. The alternative chosen for *sample F* corresponds to the one reported in the literature, where ODTMOS was added together with tetraethoxysilane (TEOS), as silica precursor [29]. A first conclusion is that homogeneous dispersions were formed when the final pH is 7 ÷ 7.4 provided by an equimolecular OLA/OLANa mixture (Figure 4).

In Figure 4, we have the modification of pH depending on the ODTMOS fraction. The increase of the ODTMOS fraction causes the increase of the content of saturated long chains and of silica from silane. The decrease of the pH can be due the increase of H^+^ ion concentration from the newly obtained hybrids. The modification of the pH value is not very significant and does not exceed the usual range of 7.1–7.4 recorded for an OLA/OLANa complex as in [32].

The inset picture is an additional proof of the preparation of stable dispersions, containing red-dyed olive oil. As mentioned earlier, for all the prepared systems, the dispersions were stable and homogeneously colored. The apparently homogeneous dispersions indicate the ability of oil phase encapsulation inside the OLA/OLANa system.

**Figure 5:**
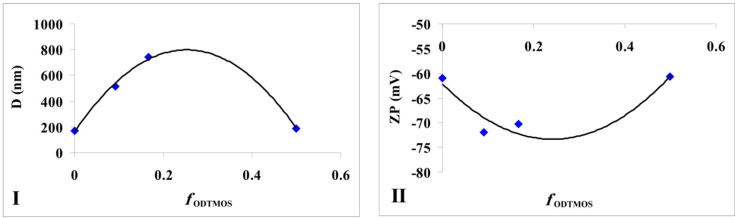
The modification of silica particles **(I**) average diameters and (**II**) zeta potential depending on the fraction of ODTMOS.

The average diameters of the aggregates formed by silica particles in the acid/soap mixture and the values of the zeta potential are presented in Table 1. The modifications that occur by varying the ODTMOS concentration were carried out at an optimal ratio of OLA/OLANa = 1/1 (*samples A*, *E*, *G* and *H*). As in the previous report that studied the influence of grafting octadecyl groups onto a silica particle’s surface [36], we could not also distinguish a monotonous modification of the particle dimensions (Figure 5I).

If in a previous study [36], the maximum value of particle diameters was obtained at a ratio of ODTMOS/TEOS = 1/10, in our case occurring at a ratio of ODTMOS/SiO_2_ = 1/5 (*sample G*). The complex behavior reflected by the morphology of hydrophobic silica particles [36], as well as the interaction of C_18_ chains (originating from ODTMOS’s condensation) with silica induced the formation of aggregates with a maximum diameter of 740 nm (*sample G*, Table 1). One can notice that, from this type of association, the zeta potential has a maximum absolute value when compared with the extremes (*samples A* and respectively *sample H*; Figure 5II).

**Figure 6:**
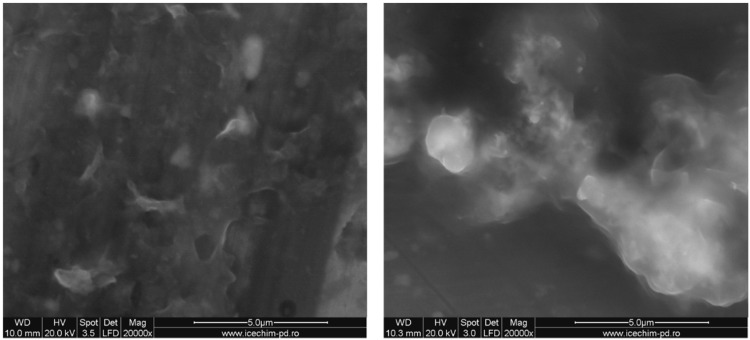
SEM pictures of (**I**) *sample A* (1/1 ODTMOS/SiO_2_) and (**II**) *sample H* (0/1 ODTMOS/SiO_2_) at 2/1 OLA/Na.

Combining several analysis methods (DLS, TEM, SEM, SANXN), Brambilla [36] presented a pattern to explain the modification of silica particles by the aggregation of octadecyl groups inserted after grafting the hydrolized ODTMOS. Thus, the silica particles changed their morphology, from a spherical to lamellar shape. The final dimensions of the composite particles are modified at a certain substitution degree due to the association in lamellar structures. In the present work, it was established that, in the case of OLA/Na = 2/1, hence at OLA/OLANa = 1/1 (Table 1), the formation of SDA vesicular structures allows the association of hybrid particles at maximum dimensions at a ratio of ODTMOS/SiO_2_ = 1/5 (*sample G*). These results are also confirmed by the SEM measurements. In Figure 6 it can be seen that, without hydrophobic substitution, the silica particles obtained in the presence of OLA/OLANa vesicles are associated in a spherical shape (*sample H*). In the presence of the octadecyl chains resulting from the reaction with ODTMOS, the aggregates adopt an associated lamellar structure, with larger sizes than the unsubstituted ones (*samples A* and *H*). The results presented in Figure 5 and Figure 6 were also verified by replacing ODTMOS with octadecyltriethoxysilane.

For ODTMOS/SiO_2_ = 1/1 molar ratio, the modification of OLA’s amount influenced the aggregates’ dimensions and the values of zeta potential. Also, the changing of the OLA/Na ratio (0.5/1 (*sample D*), 1/1 (*sample C*), 2/1 (*sample A*) and 4/1 (*sample B*)) causes modifications in the aggregates’ dimensions. Thus, for the maximum value of this ratio (*sample B*), the average particle diameter significantly increases. The excess of OLA towards the OLA/OLANa complex induces an increase of average dimensions, which is in a good agreement with the results obtained for samples without ODTMOS (Figure 2I). Although for an OLA/OLANa ratio < 1 the size of the aggregates does not exhibit great variation, the presence of ODTMOS leads to an increased association degree of the C_18_ chains from the silica hydrophobized with OLA and oleyl chains. The mixtures are all opaque, except the ones from the Figure 1-inset. The increase of the hydrophobic interactions causes modified morphologies, reflected in increased of zeta potential values with a maximum at a ratio of OLA/Na = 1/1 (*sample C*). All dispersions with a strong alkaline pH (*samples C*, *D* and *J*) are unstable. The results obtained for *sample I*, where 50% of the Na ions were neutralized with HCl, and allowed the formation of stable dispersions are very important. After the partial neutralization with HCl, OLA/Na ratio becomes 1/0.5, which is equivalent to 2/1 OLA/OLANa ratio. In the case of a more reduced neutralization (*sample J*), the final pH is 10 due to Na ion excess, and the dispersion is unstable.

Useful information’s regarding the interaction of hydrophobic groups from the synthesized dispersions, were also obtained by using DSC analysis. In good agreement with previously published results [33], when undercooled dispersions were subjected to a controlled heating, two domains of the melting temperatures were distinguished. Because the ethanol was present in the system, the water melts under 0°C (Table 1). The melting has its maximum between −2/−7 °C. The second melting interval assigned to the OLA/OLANa complex was determined around 10°C, in good agreement with another previous study [33].

**Figure 7:**
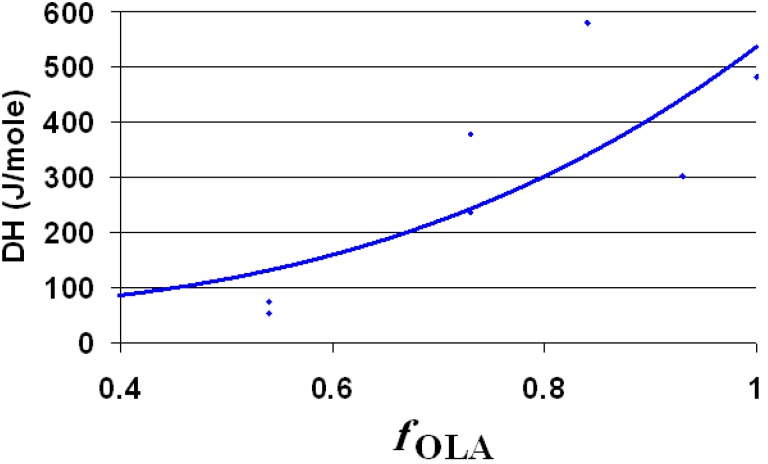
The melting enthalpy of 1 mole of hydrophobic component (OLA + ODTMOS) as function of molar ratio of OLA (*f*_OLA_ = mole OLA/mole (OLA + ODTMOS)).

Next to the OLA/OLANa couple, the dispersions contain silica and octadecyl chains (from ODTMOS). The melting enthalpy of the hydrophobic areas (around 10 °C) is strongly affected by the ratio between OLA and ODTMOS. Consequently, the enthalpy was calculated taking into account the concentration of the full hydrophobic component (OLA + ODTMOS). The melting enthalpy assigned for 1 mole of hydrophobic component (OLA + ODTMOS) is strongly affected by the molar fraction of OLA, as observed from Figure 7. The decrease of the overall melting enthalpy of hydrophobic areas presents a deviation along with the decrease of the mole fraction of OLA (Figure 7). This deviation is probably caused by the formation of some complex aggregates in which OLA is involved, due to the increase of C_18_ chains’ density.

The value of the melting enthalpy per mole for OLA (Figure 7 and Table 1) is smaller than in the case where the aqueous phase contains only the OLA/OLANa complex [26]. This modification of enthalpy is probably due to a plasticizing phenomenon caused by the presence of ethanol. An argument for this statement is the observation that the dispersion of un-modified silica (*sample H*, Table 1) is fluid, in comparison with those obtained without co-surfactant (EtOH) (Figure 2).

In solid state, the interactions between the two hydrocarbon chains (oleyl of OLA and octadecyl of ODTMOS) are also preserved. After the dispersions were dried, the maximum temperature at which the solid mixtures are thermally decomposed in air decreases with the increasing of OLA content (Table 1). This phenomenon is caused by a more reduced thermal stability of the oleyl chains, which contains double bonds. In a future study, the properties of the hybrid solids will be carefully analyzed by TGA and other methods. The data analyzed above helped to prove that, in “*green chemistry*” conditions, hydrophobic modified silica could be obtained.

In order to facilitate the thermal decomposition of the hydrocarbon chains, OLA was removed by washing it off with ammonium hydroxide and centrifugation. The lack of OLA in the washed samples was demonstrated by DSC analysis.

**Figure 8:**
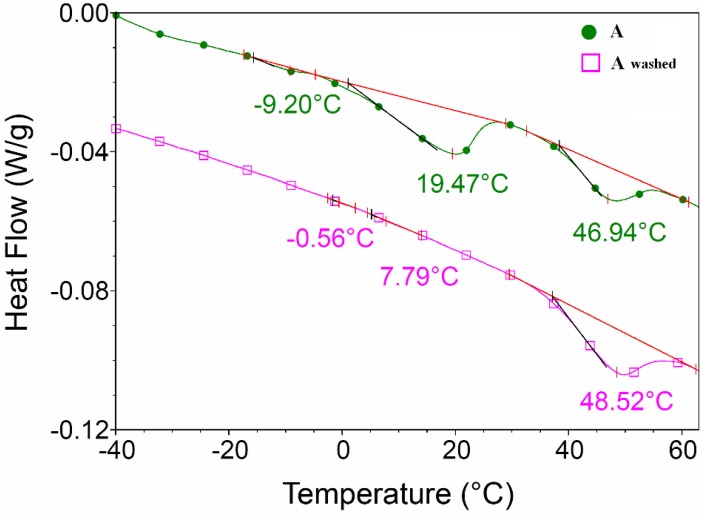
Differential scanning calorimetry (DSC) analysis of *sample A* (as synthesized and washed with ammonium hydroxide).

DSC analysis of *sample A* (as synthesized and washed, Figure 8) showed that OLA transition with maximum at 19.47 °C disappeared after OLA elimination with ammonium hydroxide.

**Figure 9:**
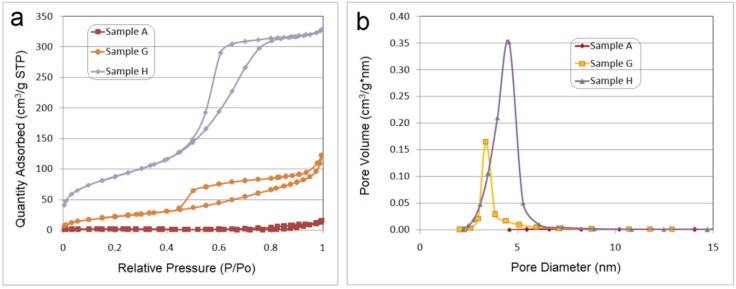
(**a**) Nitrogen isotherms for calcined *samples* (*A*, *G* and *H*); (**b**) Barrett-Joyner-Halenda (BJH) desorption dV/dD pore volume for *A*, *G* and *H samples* (OLA/ Na = 2/1; ODTMOS /SiO_2_ = 1/1, 1/5, 0/1).

Our ultimate challenge was to obtain mesoporous silica particles. In order to demonstrate the existence of nanopores in the calcinated silica, the nitrogen adsorption isotherm at −196 °C for *samples A*, *G*, *H* obtained at a substitution ratio ODTMOS/SiO_2_ = 1/1, 1/5 and 0/1 is presented in Figure 9.

The results from Figure 9, showed a similar behavior as for silica obtained with oleylamines [15]. A mutual element is the presence, beside the “ink bottle”-like mesopores, of inter-particle textural mesopores, above the relative pressure of 0.8. These textural pores are due to the aggregation of silica particles associated in lamellar aggregates, produced by the interaction of octadecyl substituents with oleyl groups from OLA/OLANa vesicular structures.

From the data presented in Table 2, it may be concluded that Brunauer-Emmett-Teller (BET) surface and pore size are significantly affected by the octadecyl chain concentration present in the pristine hybrids.

**Table 2:** Nitrogen physisorption data.

Sample No.	BET Surface Area (m^2^/g)	Pore Volume (cm^3^/g)	Pore Size (Desorption Branch) (nm)
**A**	4.2147	0.0232	15.02
**G**	84.0391	0.1890	5.25
**H**	314.73	0.5089	4.45

The increase of the ODTMOS concentration, reduced the BET surface area due to the pore blocking phenomenon. As an additional observation, after the heat treatment of *sample A*, the final silica was apparently grey, confirming the lack of thermal destruction of the trapped ocatadecyl chain.

## 3. Experimental Section

### 3.1. Materials

Oleic acid (OLA) analytical grade (UCB), octadecyltrimethoxysilane (ODTMOS) and ethanol absolute (PAN Corp., Fort Wayne, IN, USA) were used without any additional purification. Two sorts of sodium silicate (Na) were used as received: one typeof sodium silicate, containing 14.2% Na_2_O and 27.6% SiO_2_, was purchased from SC RASIN SRL (Bucharest, Romania); the other sodium silicate, with 8% Na_2_O and 26% SiO_2_, was purchased from Merck (Bucharest, Romania).

### 3.2. Synthesis Methods

#### 3.2.1. Preparation of Silica Nanoparticles from Sodium Silicate and OLA

In a reaction vessel provided with a magnetic stirrer (500 t/min), 0.8 g sodium silicate (technical) and various amounts of OLA (OLA/sodium silicate molar ratio range varies between 0.125/1 and 4/1) were introduced. Then, 20 mL of distilled water were added. The total mixture was kept under stirring for 2 h at a temperature of 40°C. The final solution was left to rest and analyzed afterwards. The resulting samples are: **1.** 0.125/1; **2.** 0.25/1; **3.** 0.5/1; **4.** 1/1; **5.** 1.5/1; **6.** 2/1; **7.** 3/1 and **8.** 4/1 OLA/Na.

#### 3.2.2. The Obtaining of Octadecyl-Silica Nanocomposites

In a reaction vessel provided with a magnetic stirrer (500 t/min), 1.6 g sodium silicate (technical) and various quantities of OLA, in order to ensure molar ratios of OLA/Na between 0.5/1 and 4/1 (see Table 1) were introduced gradually. After the addition of 40 mL of distilled water, the mixture was heated while stirring at 40 °C. After 10 min of stirring, a solution of 8 mL ethanol, 0.2 mL colored olive oil and different quantities of ODTMOS were added (see Table 1). The mixtures were stirred at 500 rpm for 4 h at 40 °C. Afterwards, they were allowed to rest, then a fraction of each sample was transferred to tightly closed glass vialsand the other part was air dried. The liquid and solid samples were subsequently used for analyses.

**Scheme 1:**
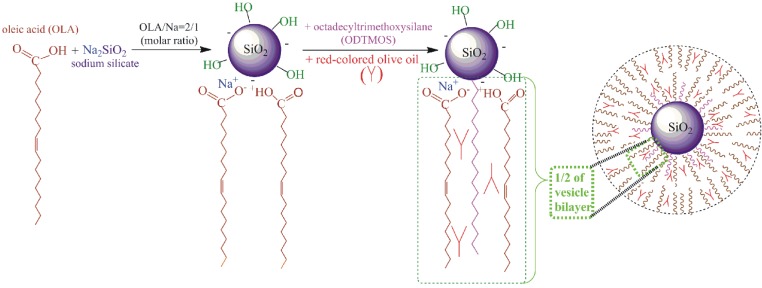
Schematic representation of the synthesis of mesoporous silica nanoparticles stabilized with an oleic acid/sodium-oleate complex starting from sodium silicate and octadecyltrimethoxysilane.

The steps to obtaining octadecyl-silica nanocomposites are shown in Scheme 1.

The excess of OLA was washed and removed by centrifugation. This process was carried out by addition of 50 mL of water that contained a sufficient quantity of ammonium hydroxide to counteract the excess of OLA. The upper aqueous phase was removed. These operations were repeated twice. The purified samples contain only silica or hydrophobic octadecyl-silica particles. After water removal, the hybrids were thermally treated to obtain mesoporous silica under a pre-established calcination program (10 min from 30 to 100 °C; 1 h at 100 °C; 20 min from 100 to 300 °C; 2 h at 300 °C; 2½ h from 300 to 500 °C; 5 h to 500 °C; 20 min from 500 to 650 °C; 1 h 650 °C; slow cooling).

### 3.3. Characterization Methods

***pH values:*** were determined with a portable pH meter from Hanna Instruments, with accurate readings from 0 to 14 pH, resolution of 0.01 pH.

***Dynamic Light Scattering (DLS) and Laser Doppler Velocimetry (LDV):*** techniques were used to determine particles size distribution and zeta potential respectively (ZetasizerNanoZS instrument Malvern Instruments Ltd., Malvern, UK). The determinations for silica-OLA samples (0.4 mL sample in 25 mL distilled water, ultrasonicated 3 min.), were performed at 50 °C. For the hybrid silica-OLA-ODTMOS samples, 0.2 mL of the obtained sample were diluted in 25 mL distilled water and ultrasonicated for 10 min. Samples were equilibrated for 10 min before being analyzed at 50 °C. The size distribution by intensity was considered for the average diameter evaluation.

***Differential scanning calorimetry (DSC):*** thermographs were recorded with DSC Q2000 (TA Instruments, Zellik, Belgium) in the following conditions: Cooling Unit LNCS; Purge Gas: Helium 30 mL/min; Pan: Hermetic Aluminum; Sample Size: 9–11 mg; Method: Ramp 10 °C/min (from room temperature to −43 °C; Isothermal for 1 min; Ramp 5 °C/min to 60 °C; Isothermal for 1 min; Ramp 5 °C/min to −43 °C.

***N_2_ adsorption-desorption:*** measurements were carried out on a volumetric adsorption analyzer Micromeritics ASAP 2020 at the liquid nitrogen temperature (−196 °C).

Diluted samples (from DLS analyses) were casted on aluminum stubs. After drying at ambient temperature, the samples were investigated by Scanning Electron Microscopy (SEM-FEI Quanta 200, Eindhoven, The Netherlands), without covering. The images were acquired in low vacuum (1 torr) using Large Field Detector.

## 4. Conclusions

Starting from vesicular structure, consisting of oleic acid (OLA) and its sodium salt (OLANa) and using soft green chemistry conditions, silica particles stabilized in water were obtained for the first time. Depending on the molar ratio of OLA/OLANa, the dimensions of associated silica particles modify. Thus, the average diameter of the resulting particles significantly increases at a molar ratio of OLA/OLANa > 2/1.

By grafting preformed silica particles with octadecyl groups from ODTMOS, aqueous stable dispersions, able to solubilize the hydrophobic colored olive oil, were obtained. At a molar ratio of ODTMOS/SiO_2_ = 1/5, and an OLA/Na = 2/1 molar ratio, the largest sizes of the hydrophobic silica particles were obtained. These particles contain silica with pores bearing medium diameters of 3.5 nm, but also with an important percentage of interparticle textural mesopores. The synthesis conditions are ecological, according to green chemistry, and the application of raw materials gives them a biocompatible nature.

These preliminary data allow us to believe that by using renewable vegetable raw materials (for example, OLA or other unsaturated fatty acids) and sand reserves, the conditions to obtain mesoporous silica are satisfied. Nanometric pores, as well as the associated hydrocarbon chains, allow the stabilization of natural products, like olive oil in aqueous dispersions. After this first step, forthcoming studies will also highlight other aspects regarding the properties of these biodegradable and ecologic nanometric dispersions.

## Supplementary Materials

The following are available online at http://www.mdpi.com/2079-4991/6/1/9/s1.
