# Supplementary material for: Aqueous Dispersions of Silica Stabilized with Oleic Acid Obtained by Green Chemistry
Source: Nanomaterials (Basel). 2016 Jan 5;6(1):9. doi: 10.3390/nano6010009 (PMC5302543; doi:10.3390/nano6010009)
Supplement: Supplementary file 1 [file nanomaterials-06-00009-s001.pdf]

Supplementary Material

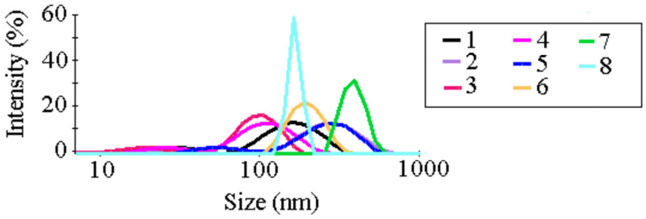

Figure S1. Size distribution by intensity for the dispersions prepared with different quantities of OLA and a fixed amount of sodium silicate (**1.** 0.125/1; **2.** 0.25/1; **3.** 0.5/1; **4.** 1/1; **5.** 1.5/1; **6.** 2/1; **7.** 3/1 and **8.** 4/1 OLA/Na).

© 2016 by the authors; licensee MDPI, Basel, Switzerland. This article is an open access article distributed under the terms and conditions of the Creative Commons by Attribution (CC-BY) license (http://creativecommons.org/licenses/by/4.0/).
